# In pursuit of a flawless aphrodite: paving the way to scarless oncoplastic breast surgery

**DOI:** 10.1186/s40880-019-0422-4

**Published:** 2019-12-04

**Authors:** Liling Zhu, Shunrong Li, Luyuan Tan, Xiaolan Zhang, Jiannan Wu, Fengxi Su, Kai Chen, Erwei Song

**Affiliations:** 1 Guangdong Provincial Key Laboratory of Malignant Tumor Epigenetics and Gene Regulation, Sun Yat-Sen Memorial Hospital, Sun Yat-Sen University, Guangzhou, 510120 Guangdong P.R. China; 2 Breast Tumor Center, Sun Yat-sen Memorial Hospital, Sun Yat-sen University, Guangzhou, 510120 Guangdong P.R. China; 3 Department of Breast Surgery, Guangzhou Concord Cancer Center, Guangzhou, 510045 Guangdong P.R. China; 4 Sufengxi Clinic, Guangzhou, 510000 Guangdong P.R. China

## Introduction

Breast cancer is the most commonly diagnosed malignancy in women worldwide [1, 2] and in the twentieth century, mastectomy was the primary surgical treatment for breast cancer patients. Advances in breast-conserving surgery (BCS) propelled a change in treatment rational from “maximum tolerable” to “minimum effective” therapy. Oncoplastic breast surgery aims to restore the shape of the breast and has been widely adopted since the past decade. Although the cosmetic outcome has been significantly improved, the scar remaining on the surgeried breast skin is still a major pitfall that urges urgent consideration. In this editorial, we review a series of techniques that can be incorporated in oncoplastic breast surgery to minimize scarring, signifying the beginning of an era for scarless oncoplastic surgery.

## A brief history of breast surgical oncology

The Halstedian theory proposed that breast cancer developed in situ while its metastasis developed in contiguous patterns via the lymphatic system. If the lymph nodes that act as barriers were compromised, tumor cells were to then move into blood vessels, causing distant metastasis [3]. Based on this theory, (modified) radical mastectomy was proposed as the standard surgical treatment for breast cancer patients throughout the first half of the twentieth century. The rationale behind this proposal was that the eradication of tumor cells was more likely with more expansive resection of the surgical region. In the 1960s, Bernard Fisher conducted a series of basic and translational studies which suggested that tumor cells had no orderly pattern of metastasis. Breast cancer cells might spread into the blood vessels at an early stage even in the absence of lymph node metastasis [4, 5]. According to Fisher, the occurrence of distant metastasis is determined by the complex host-tumor interactions (alternative hypothesis) [6]. Based on this theory, BCS was proposed and has been proven to be oncologically safe in a series of multicenter randomized controlled trials [7–9]. BCS can significantly improve the cosmetic outcome as well as the quality of life (QoL) of breast cancer patients. However, due to the amount of tissues removed, the breast that received traditional BCS might not be symmetric to the contralateral one, which compromises the cosmetic outcomes in some patients. For example, Clough et al. [10] reported that “bird’s beak” deformity is usually observed in breast cancer patients with tumors located in the lower pole of the breast. Thus, oncoplastic breast surgery that integrates techniques of breast surgical oncology with plastic surgery was proposed in order to improve the cosmetic outcome of breast cancer treatment. Oncoplastic breast surgery includes two different approaches: volume displacement and volume replacement [11]. The volume displacement approach utilizes glandular reshaping, tissue approximation or reduction mammoplasty to make up for defects resulting from tumor extirpation. Clough et al. [10] had proposed oncoplastic BCS techniques in a quadrant per quadrant atlas, which were shown to be safe and had been widely used in clinical practices [12, 13]. On the contrary, the volume replacement approach utilizes silicone implants or autologous tissue flap to reconstruct a new breast.

Over the past century, the overall trends of breast cancer surgery were not only to reduce the resection region (mastectomy to BCS) but also to focus more on its cosmetic outcomes (BCS to oncoplastic surgery). Oncoplastic surgery is the current standard-of-care for the surgical treatment for early-stage breast cancer patients worldwide. However, oncoplastic surgery is sometimes associated with significant scarring, which may be a painful and undesirable remembrance for breast cancer patients about their traumatic experience. To conquer the final miles of this ongoing enhancing post-cosmetic-cancer recovery marathon, we hereby propose a scarless oncoplastic breast surgery approach.

## Definition

The scarless oncoplastic breast surgery is defined as an oncoplastic surgery that uses modified techniques to minimize scarring on the breast. This scarless strategy can be applied in both the volume displacement and volume replacement approach.

## Scarless strategy in volume displacement approach

**Fig. 1:**
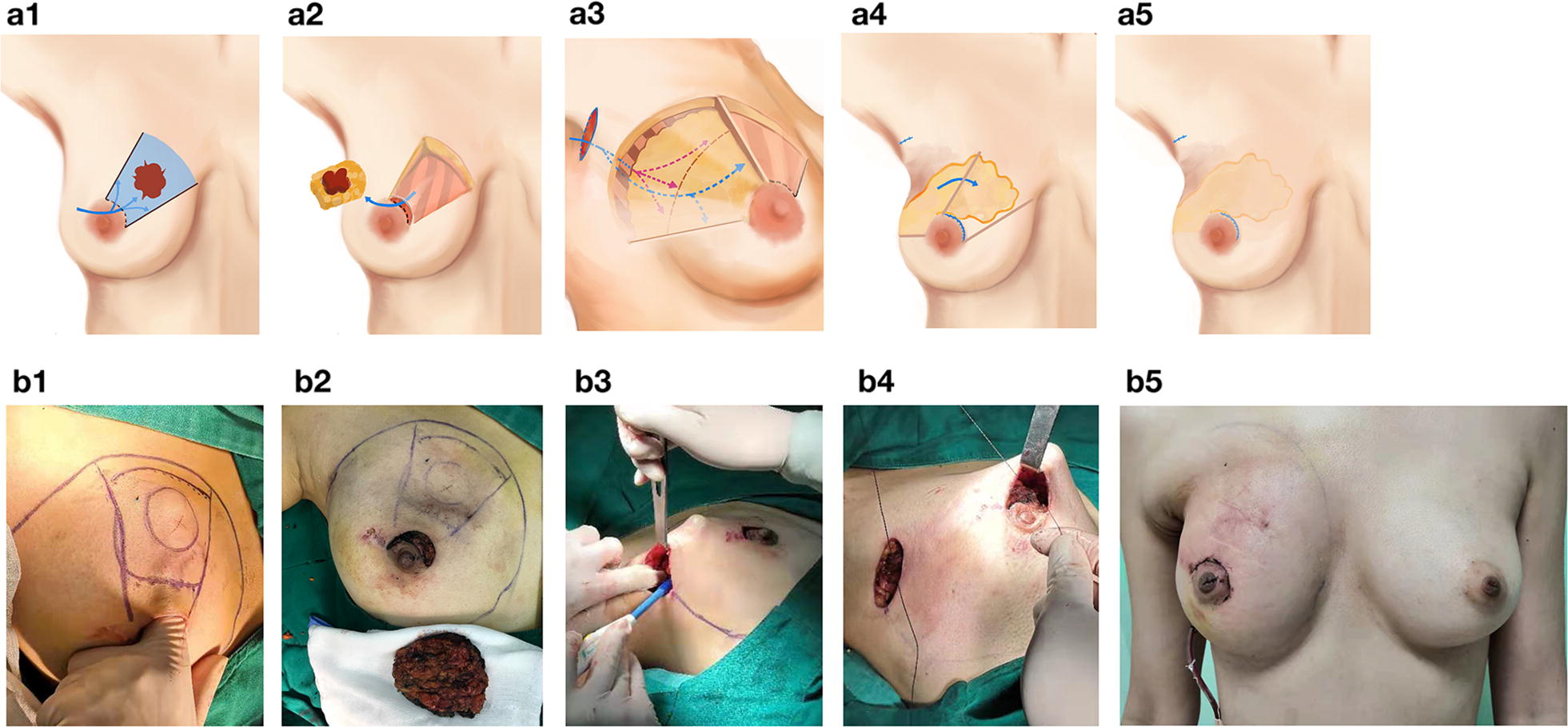
Atlas of scarless oncoplastic breast-conserving surgery. **a1**, **b1** The location of the tumor, as well as the planned excision region, were marked on the skin of the breast, and a semi-peri-nipple-areolar-complex incision was done to remove the tumor-containing specimen. **a2**, **b2** A lighted retractor is useful for the excision. The surgical margin assessment was performed as a standard-of-care practice. **a3**, **b3** The superficial layer of the upper outer quadrant of the breast was undermined through a semi-peri-nipple-areolar-complex incision and the axillary incision that was made for sentinel lymph node biopsy. The corresponding retro-mammary space was not undermined completely. Usually, one-third to half of the retro-mammary space is undermined. **a4**, **b4** The upper outer quadrant of the breast tissues were then rotated medially into the cavity. **a5**, **b5** The scar of this procedure can be minimized with satisfactory cosmetic outcome. The atlas (**a1**–**a5**) was produced by Dr. Yinuo Huang; the surgery was performed by Dr. Erwei Song and Dr. Kai Chen; the photographs were provided by Dr. Kai Chen, with consent obtained from the patient

**Fig. 2:**
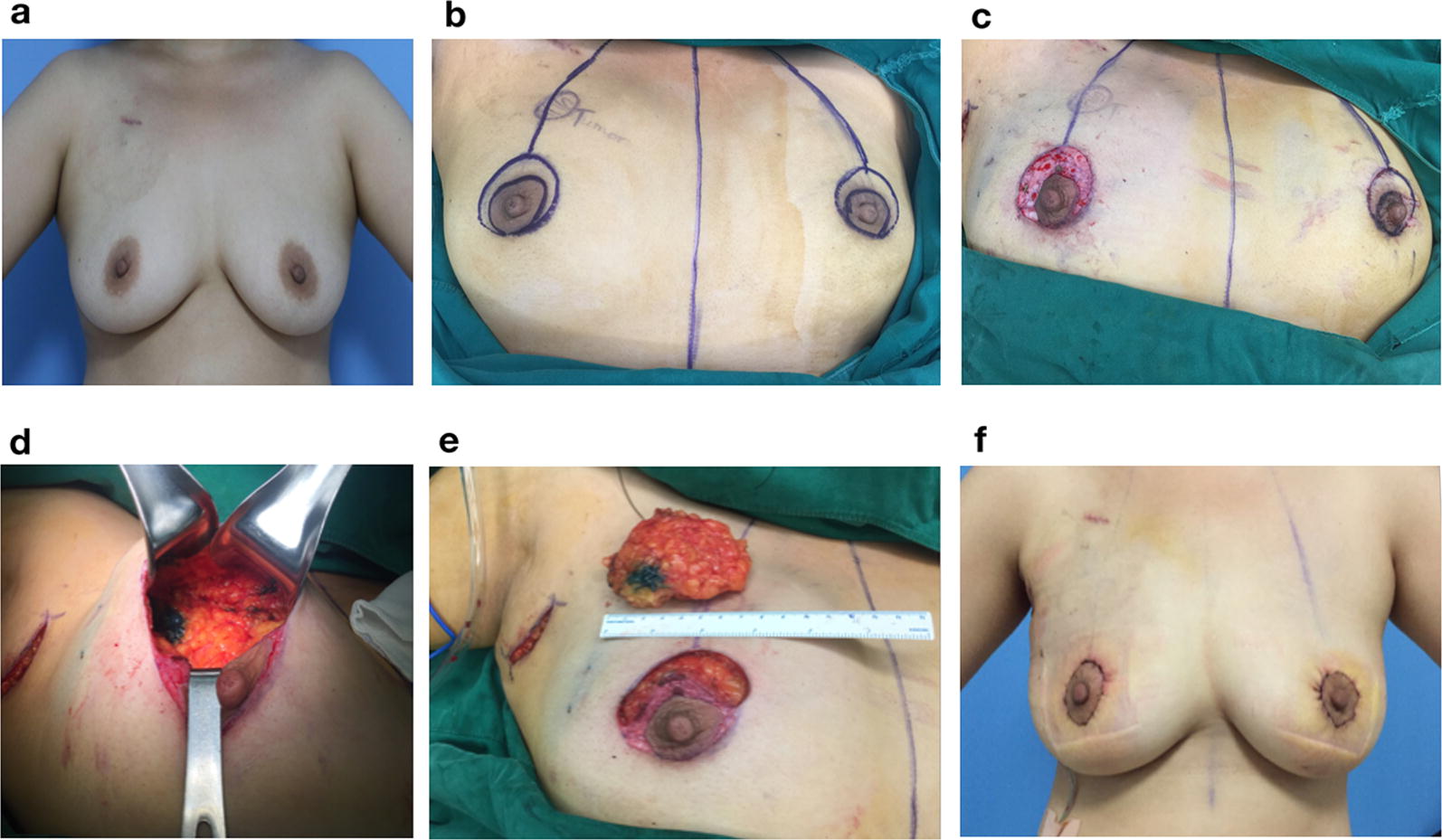
Atlas of round-block technique for oncoplastic breast-conserving surgery **a**, **b** Pre-operation marking of the incision, as well as the peri-NAC area for de-epithelialization. **c** De-epithelialization of the planned area. **d**, **e** Removing the tumor-containing specimen, and re-approximate the residue breast glands. **f** Post-operative photograph of the patient. The surgery was performed by Dr. Fengxi Su and Dr. Jiannan Wu with consent obtained from the patient

Clough et al. [10] reported an excellent summary of oncoplastic BCS in different quadrants. However, most of the approaches might lead to significant scarring on the breast. Scarless oncoplastic BCS can be used to minimize surgical scars for tumors located in different quadrants of the breast. For tumors located in the upper inner quadrant or upper pole of the breast, a rotation glandular flap can be used with a semi-nipple-areolar incision to minimize the scarring (Fig. 1). Our approach is different from the one reported by Massey et al. [14]. For tumors located at the upper outer quadrant, endoscopic-assisted BCS can be applied, as Takahashi et al. [15] previously reported, describing that this procedure would simply leave a semi-nipple-areolar incision scar on the breast. Alternatively, the round-block technique [16] can be considered for upper quadrant breast tumors (Fig. 2).

## Scarless strategy in volume replacement approach

Immediate silicone implant-based breast reconstruction after nipple-sparing mastectomy (NSM) is an important volume replacement strategy. We had previously reported the oncological safety of NSM [17]. Several types of incisions can be used in NSM, including periareolar incision with lateral extension, italic S incision on the upper outer quadrant, and inframammary fold incision [18]. However, all of these incisions might again leave significant scars on the breast. With the help of endoscopic technique, NSM can be performed through a single axillary incision made for axillary surgery, which is able to significantly minimize the scarring on the breast (endoscopic nipple sparing mastectomy, ENSM technique). Lai et al. [19] reported their preliminary results of ENSM, suggesting its oncological safety while Du et al. [20] reported in a prospective non-randomized study that patients who underwent ENSM were more satisfied with their cosmetic outcomes as compared to those who underwent the traditional BCS.

**Fig.3:**
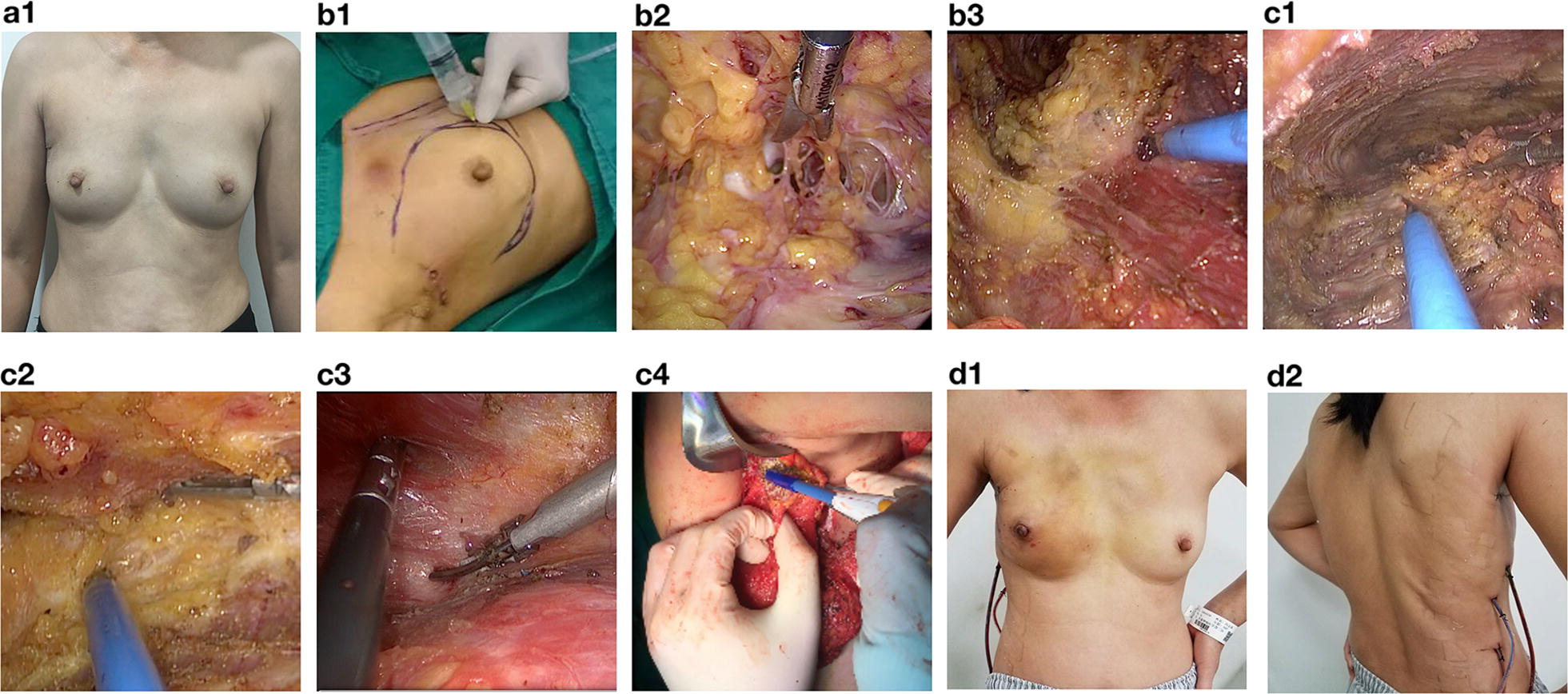
Endoscopy assisted nipple-sparing mastectomy to minimize the scar. ENSM with immediate breast reconstruction using pedicled latissimus dorsi muscle flap (LDM flap). **a1** Pre-operative photograph of the patient. **b1** 200–250 cc of lipolysis fluid was injected subcutaneously, and liposuction was performed 5 min after the injection. **b2** After liposuction, single port endoscopy was done and the subcutaneous space was established. Cooper’s ligament and breast ducts under the NAC were identified. **b3** The retro-mammary space was undermined. **c1** Lateral border of the LDM flap was elevated. **c2** The subcutaneous layer was undermined, and special attention was paid to preserve as much fatty tissues as possible. **c3** The LDM flap was elevated. **c4** The thoracodorsal nerve was dissected after the LDM flap was isolated. **d1**, **d2** Post-operative photographs showing no scars on the breast and the back. The surgery was performed by Dr. Erwei Song and Dr. Kai Chen. The photographs were provided by Dr. Kai Chen, with consent obtained from the patient

**Fig. 4:**
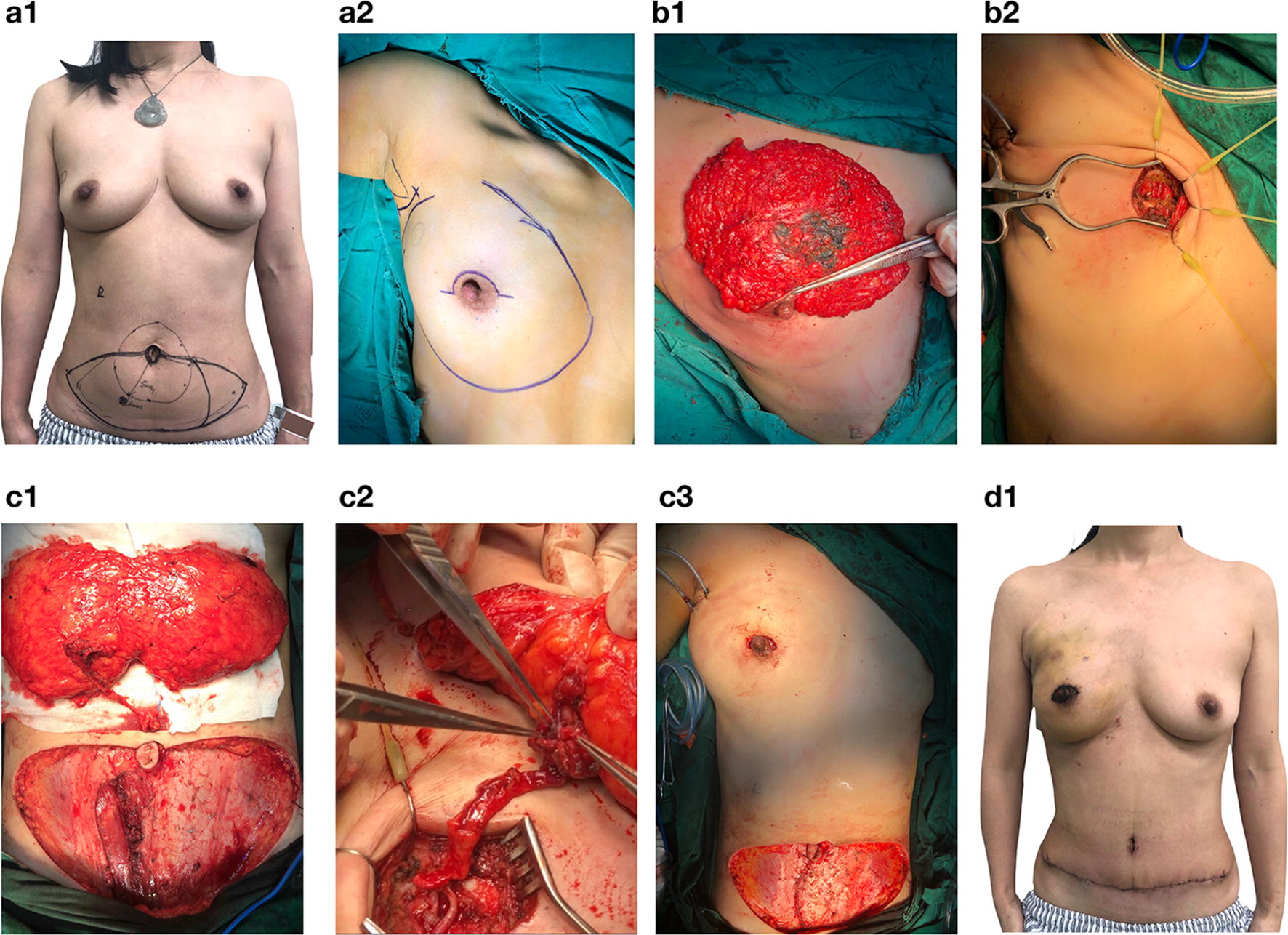
Nipple sparing mastectomy(NSM) with the burried DIEP flap breast reconstruction. DIEP flap reconstruction after NSM with the peri-NAC incision. **a1**, **a2** Pre-operation marking of the incision, the contour of the breast, and the flap. **b1** NSM was performed, and the breast tissue was removed via the peri-NAC incision. **b2** The second to third intercostal space was exposed. **c1**, **c2** The DIEP flap was harvested, and the anastomoses of the right deep inferior epigastric vessels to the right internal mammary vessels were performed. **d1** Post-operative photograph of the patient. The surgery was performed by Dr. Shunrong Li, Dr. Liling Zhu and Dr. Erwei Song. The photographs were provided by Dr. Liling Zhu, with consent obtained from the patient

There are a variety of options available for immediate breast reconstruction after ENSM besides silicone implant. Satake et al. [21] reported a novel technique that uses multistage fat grafting after ENSM for breast reconstruction. We had also confirmed the efficacy and feasibility of latissimus dorsi muscle (LDM) flaps after ENSM [22] (Fig. 3). Deep inferior epigastric perforator (DIEP) flap is a free flap that has much more advantages over pedicle flaps for breast reconstruction. To further improve the cosmetic outcome, NSM that preserve the nipple-areola complex (NAC) can be used for DIEP flap breast reconstruction. Fujimoto et al. [23] reported that NSM was oncologically safe for DIEP flap breast reconstruction as long as their respective pathology examinations confirmed the absence of tumor cells in the subareolar tissue. However, a skin paddle that serves as a window for post-operative monitoring of arterial ischemia or venous congestion of the flap, is often needed even if the NSM technique was used [24]. To significantly reduce the scar on the surgeried breast, Frey et al. [25] placed an implantable Doppler probe around the arterial and/or venous anastomoses, so as to omit the need of a skin paddle. In a retrospective study, they reported that the flap failure rates (2.0% vs. 2.3%) and re-operative rates (6.0% vs. 4.7%) were similar between patients with and without a skin paddle after DIEP flap breast reconstruction with NSM [25]. In fact, skin paddles and/or implantable Doppler devices are not necessary for experienced surgeons. Levy et al. [26] “buried” the flap without any skin paddles after DIEP flap breast reconstruction with NSM, and performed the surveillance by transcutaneous Doppler and clinical observation, e.g. drainage, skin color and breast volume. Their clinical outcomes were satisfactory. Similarly, we routinely performed DIEP flap breast reconstruction with NSM without any skin paddles or implantable Doppler surveillance, and the patients’ satisfaction is good (Fig. 4).

## Unsolved riddles for the future

Although there have been advancements in improving the post-surgery cosmetic outcomes of breast cancer patients, the scar left, no matter the age of the patient, still have repercussions on the patient QoL [27, 28]. There are still several unanswered issues that still need to be further examined before scarless oncoplastic breast surgery can be widely incorporated into clinics or guidelines. First, the eligibility of patients for each scarless oncoplastic surgery needs to be further addressed. For instance, ENSM with liposuction might not be performed for large tumors, or tumors in close proximity to the subcutaneous layer. But the detailed criteria needs further investigations. Magnetic resonance imaging (MRI) examination might be needed to screen for eligibility prior to ENSM. Second, the oncological safety and cosmetic outcomes should be fully assessed in prospective, multicenter, randomized clinical trial. The underlying oncological safety is considered as the most important prerequisite for scarless oncoplastic surgery. Evaluation methods using standardized criteria such as relapse-free survival, disease-free survival and overall survival can be used, however, the basis for evaluating its cosmetic outcomes remained varied among different studies. A standardized method like Breast-Q [29] is needed to ensure that the assessment of cosmetic outcomes is comparable between institutions. Third, the development of novel biomedical techniques such as robotic surgery may further facilitate scar less operative strategies. Post-operative treatments such as taping, silicone gel and moisturizing have been demonstrated as optional prevention methods for hypertrophied scar formation [30]. Other treatments such as oils, lotion, laser therapy, massage therapy, radiotherapy, and more, are still being investigated for their potential as post-operative treatments to minimize scar formation [30].

Over the past century, surgical treatment of breast cancer has evolved towards a better cosmetic outcome, from mastectomy to BCS, and now to oncoplastic surgery. Improving cosmetic outcomes have been found to be associated with improved QoL for breast cancer survivors. Although oncoplastic surgery is successful in restoring the shape of the original breast, the scars are the final mile of this marathon that remain to be solved for optimizing the oncoplastic treatment of breast cancer.

## Abbreviations

BCS: breast-conserving surgery
QoL: quality of life
NSM: nipple-sparing mastectomy
ENSM: endoscopic nipple sparing mastectomy
LDM: latissimus dorsi muscle
DIEP: deep inferior epigastric perforator
NAC: nipple-areola complex
MRI: magnetic resonance imaging
